# Relationship between renal tissues phospholipase A2 receptor and its serum antibody and clinical condition and prognosis of idiopathic membranous nephropathy: a meta-analysis

**DOI:** 10.1186/s12882-019-1638-x

**Published:** 2019-12-02

**Authors:** Dan Dong, Ting-ting Fan, Ying-ying Wang, Lu Zhang, Li Song, Li Zhang

**Affiliations:** 1 Department of Nephrology, First Hospital of Jilin University, 71 Xinmin Street, Changchun, Jilin, 130021 People’s Republic of China; 2 Department of Nephrology, Binzhou People’s Hospital, Binzhou, Shandong People’s Republic of China; 3 Department of Nephrology, Jining No.1 People’s Hospital, Jining, Shandong People’s Republic of China

**Keywords:** PLA2R, Anti-PLA2R antibody, Idiopathic membranous nephropathy, Meta-analysis

## Abstract

**Objective:**

To investigate the correlation of M-type phospholipase A2 receptor (PLA2R) expression and serum anti-PLA2R antibody with the clinical parameters and prognosis of patients with idiopathic membranous nephropathy (IMN).

**Methods:**

A literature search for relevant original articles published between January 2009 and October 2019 was conducted on domestic and foreign databases. RevMan 5.3 software was used for meta-analysis.

**Results:**

Eighteen studies were included in this meta-analysis. There were 1235 anti-PLA2R antibody-positive and PLA2R-positive patients, and 407 serum anti-PLA2R antibody-negative and PLA2R-negative patients. Compared with negative group, patients in the serum PLA2R antibody -positive group had lower serum albumin [SMD = -1.11, 95% CI (− 1.82, − 0.40), *P* < 0.00001], higher age [MD = 2.71, 95% CI (1.94, 3.48), *P* < 0.00001], and lower estimated glomerular filtration rate (eGFR) [MD = -10.34, 95% CI (− 12.09, − 8.60), *P* < 0.00001]; no significant between-group difference was observed with respect to 24-h urine protein and serum creatinine. However, no significant difference was observed between renal tissues PLA2R -positive and -negative groups with respect to serum albumin, eGFR, serum creatinine, and 24-h urine protein. Remission rate in the serum anti-PLA2R antibody -positive group was lower than that in the -negative group [OR = 0.41, 95% CI (0.28, 0.61),*P* < 0.00001]; however, no significant between-group difference in this respect was observed between the renal tissue PLA2R-positive and -negative groups. In the serum anti-PLA2R antibody -positive group, the higher titer subgroup had lower remission rate [OR = 0.19, 95% CI (0.07, 0.55),*P* = 0.002]. No significant difference was observed between anti-PLA2R antibody -positive and -negative groups with respect to adverse events. Serum anti-PLA2R antibody titer did not affect the adverse event rate.

**Conclusion:**

As compared to PLA2R, serum anti-PLA2R antibody is more closely related with IMN disease progression.

## Background

Membranous nephropathy (MN) refers to a group of kidney diseases characterized by thickened glomerular basement membrane (GBM) and deposition of immune complexes on the GBM. According to the etiology, MN is classified into two types: idiopathic membranous nephropathy (IMN) and secondary membranous nephropathy (SMN) [1]. Studies have shown that about 40% of patients with IMN eventually develop end-stage renal disease (ESRD) [2]. Phospholipase A2 receptor (PLA2R) is a protein with a relative molecular weight of 180–200 kD. It is expressed on the membrane of human podocytes and belongs to the family of mannose receptors [3]. Till date, two subtypes of PLA2R have been found, namely M-type and N-type [4]. PLA2R is widely distributed in various organs of the human body, such as lung, kidney, and placenta [5]. In the kidney, M-type PLA2R is mainly expressed in the cytoplasm and membrane of the podocytes [6]. In 2009, Beck et al. detected the anti-PLA2R antibody (anti-PLA2R Ab) in the serum of adult IMN patients and used it as a specific marker of IMN [7]. Since then, a series of studies [8–12] have demonstrated that renal expression of PLA2R and serum anti-PLA2R antibodies can be used for diagnosis, and prognostic assessment of patients with IMN. However, no systematic review has evaluated the relationship of PLA2R and serum anti-PLA2R antibodies with IMN disease. In the present study, we performed a meta-analysis of data from relevant published clinical studies to evaluate this relationship.

## Methods

### Eligibility criteria

Studies that qualified the following criteria were included: (1) study type: cohort study; (2) Patients were diagnosed as idiopathic membranous nephropathy by renal biopsy; (3) The exposure factors: the study group was positive for serum PLA2R antibody or kidney tissues were PLA2R -positive, and the control group was serum PLA2R antibody -negative or renal tissues PLA2R -negative. (4) primary endpoint was complete remission (as defined in the included study) or partial remission (as defined in the included study) at the end of follow-up; secondary endpoint was adverse events such as renal injury, continuous proteinuria, progression to ESRD, disease palindromia, and death.

The exclusion criteria were: (1) patients with SMN; (2) duplicate publications; (3) literature reviews and animal experiments; (4) studies that had a follow-up time of < 6 months.

### Data sources and search strategy

Relevant studies published between January 2009 and October 2019 which investigated the relationship of PLA2R and its antibodies with the clinical parameters and prognosis of IMN were retrieved from Pubmed, EMBASE, MEDLINE, China Biomedical Literature Database, Chinese Journal Full-text Database, and Wanfang Database. We adopted joint search of free words and subject words. Chinese search terms included: glomerulonephritis, membranous nephropathy, M-type phospholipase A2, and cohort studies. English search terms included: “Glomerulonephritis, Membranous” [Mesh], “Receptors, Phospholipase A2” [Mesh], “Cohort Studies” [Mesh].

### Document retrieval process and results

A total of 683 documents were retrieved on initial literature search. The title and abstracts were screened independently by two reviewers to eliminate irrelevant articles. Full text of the remaining documents was reviewed against the inclusion and exclusion criteria. In case of any disagreement between the two reviewers, the decision was taken by consensus or by a third reviewer.

**Fig. 1:**
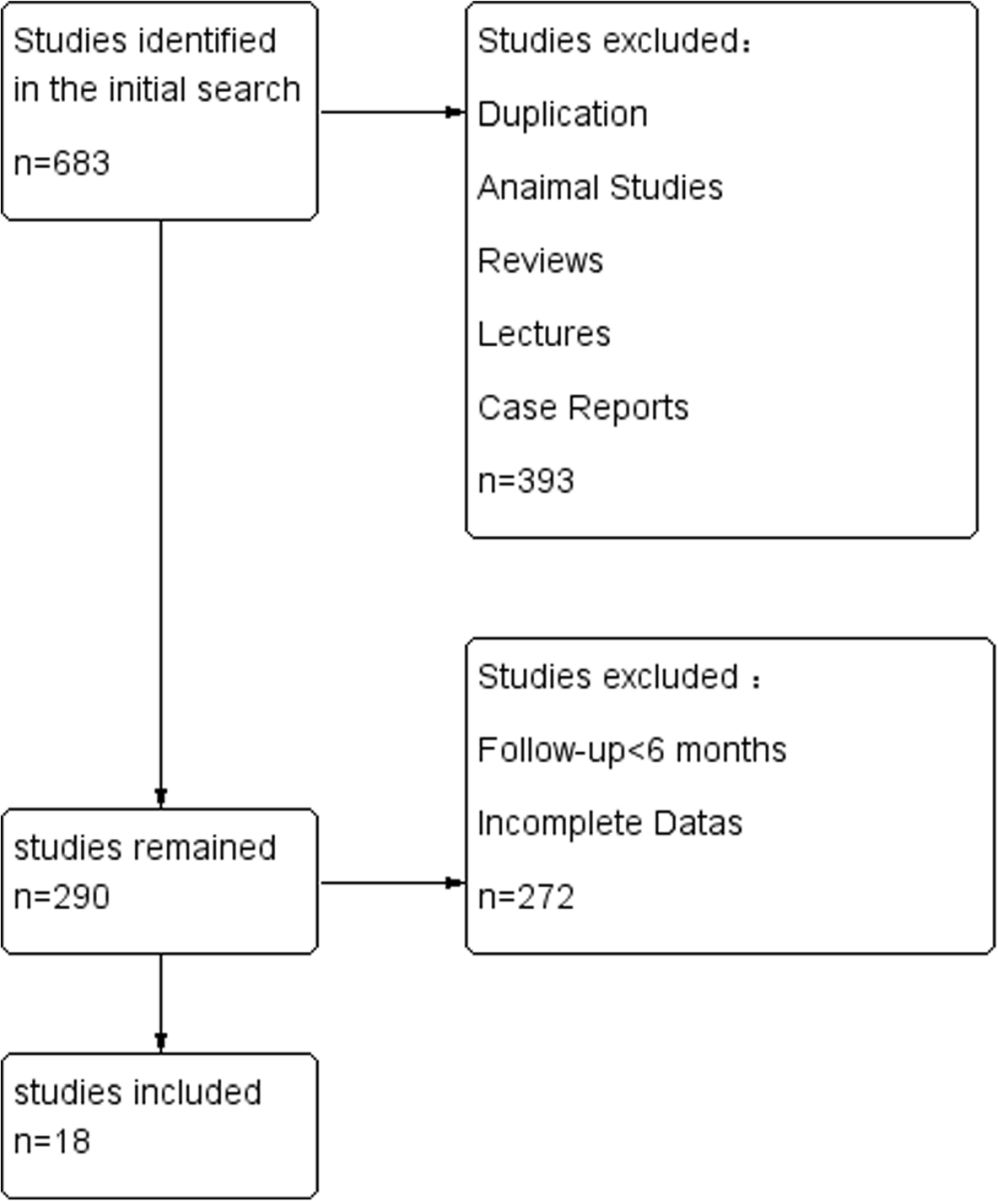
Schematic illustration of the literature search and study selection criteria

Finally, only 18 relevant publications were selected for this meta-analysis [8, 10, 11, 13–27]. These studies included 1235 PLA2R-positive and serum anti-PLA2R antibody-positive patients and 407 PLA2R-negative and serum PLA2R antibody-negative patients. A schematic illustration of literature search and results is shown in Fig. 1. The basic characteristics and results of quality assessment of the included studies are shown in Table 1. We measured serum creatinine, serum albumin, 24 h urine protein, eGFR to reflect the impairment of renal function in patients before treatment in anti-PLA2R antibody-positive, anti-PLA2R antibody-negative, kidney tissues PLA2R -positive and -negative groups. The remission rate and adverse prognosis was used to reflect the prognosis after treatment in anti-PLA2R antibody, renal tissue PLA2R -positive and -negative groups.

**Table 1:** Baseline characteristics of cohorts in all included studies

Studies	area	PLA2R/anti-PLA2R (+/-)	Testing location	Testing Method	Follow-up (month)	NOS scores
Wang LJ 2016	China	40/16	renal tissues	IFA	12	8
Liu H 2016	China	56/11	renal tissues	IFA	>12	8
Pourcine F 2017	France	69/8	serum	ELISA	168	8
Ramachandran R 2016	India	94/20	serum	ELISA	12	7
Beck LH 2011	Canada	25/10	serum	WB	24	8
Wang J 2017	China	78/13	renal tissues	IFA	15	7
Bech AP 2014	Hollan	34/14	serum	ELISA	60	8
Zhou GY 2017	China	40/16	serum	ELISA	6	7
Qin W 2011	China	49/11	serum	ELISA	>12	5
Oh YJ 2013	Korea	69/31	serum	WB	30	8
Kim YG2015	Korea	41/52	serum	ELISA	24	7
Ruggenenti P 2015	France	81/20	serum	ELISA	12	6
Timmermans SA 2015	Hollan	65/8	serum	IFA	11.3	6
Hofstra JM 2012	Europe	82/28	serum	IFA、ELISA	54	6
Liang Y 2017	China	42/37*	serum	ELISA	12	7
Huang J 2017	China	29/11	serum	IFA	>12	7
Zhang D 2018	China	164/28	serum	ELISA	>13.6	7
Li Q 2018	China	177/73	serum	ELISA	>6	7

Note:*IFA* indirect immunofluorescence assay, *ELISA* enzyme-linked immunosorbent assay, *WB* Western blotting, * High titer/Low titer

### Quality assessment

The Newcastle-Ottawa Scale (NOS) for quality assessment of non-randomized controlled studies was used to evaluate the quality of the included studies, including aspects such as selection of subjects, comparability between groups and measurement of results. The full score is 9, and a study received more than 5 was quality assessed. The quality assessment was performed independently by two reviewers; disagreements, if any, were resolved by consensus or by involvement of a third reviewer.

### Statistical analysis

Meta-analysis was performed using RevMan 5.3 software. Mean difference (MD) and standardized mean difference (SMD) were used for continuous variables, while relative risk (RR) or odds ratio (OR) was used as effect size for categorical variables; 95% confidence intervals were calculated for both. Heterogeneity among the included studies was assessed using *I*^2^ statistic. In the absence of significant heterogeneity among the included studies (*P* > 0.1, *I*^*2*^ < 50%), a fixed effect model was used for meta-analysis; in case of significant heterogeneity (*P* < 0.1, *I*^*2*^ > 50%), a random effect model was used for meta-analysis. Publication bias was assessed by Egger’s test and Begg‘s test using Stata13.0 software.

## Results

## Comparison of serum albumin levels in anti-PLA2R antibody-positive, kidney tissues PLA2R -positive and -negative groups

**Fig. 2:**
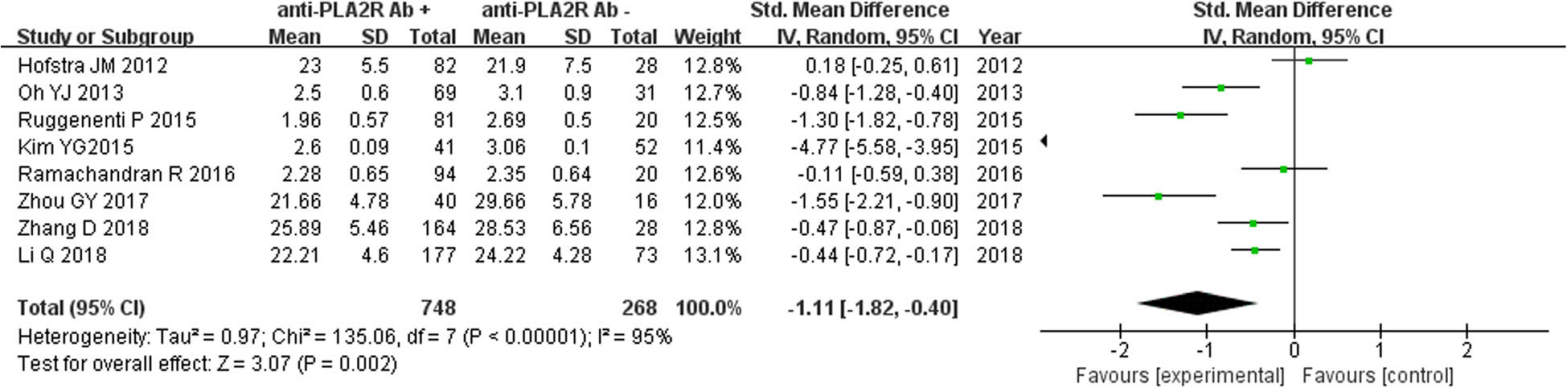
Meta-analysis of serum albumin levels: serum anti-PLA2R antibody-positive group versus -negative group

Eight studies had reported serum albumin levels in IMN patients disaggregated by serum anti-PLA2R antibody-positive (*n* = 748) and -negative groups (*n* = 268). Owing to significant heterogeneity among the studies (*P* < 0.00001, *I*^*2*^ = 95%), a random effect model was used for the meta-analysis. Serum albumin level in anti-PLA2R antibody-positive patients was significantly lower than that in anti-PLA2R antibody-negative patients [SMD = -1.11, 95% CI (− 1.82, − 0.40), *P* = 0.002] (Fig. 2).

**Fig. 3:**
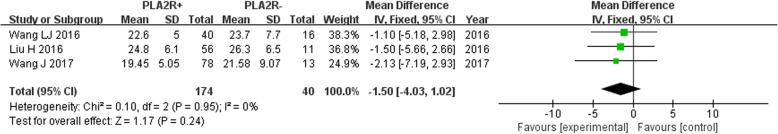
Meta-analysis of serum albumin levels: renal tissue PLA2R-positive group versus -negative group

Three studies had reported serum albumin levels in IMN patients disaggregated by renal tissue PLA2R-positive and -negative groups. Owing to the lack of significant heterogeneity among the studies (*P* = 0.95, *I*^*2*^ = 0%), the fixed effect model was used for the analysis. The results showed no significant between-group difference with respect to serum albumin levels [MD = -1.50, 95% CI (− 4.03, − 1.02), *P* = 0.24] (Fig. 3).

### Comparison of age between renal tissue PLA2R-positive, anti-PLA2R antibody-positive and -negative groups

**Fig. 4:**
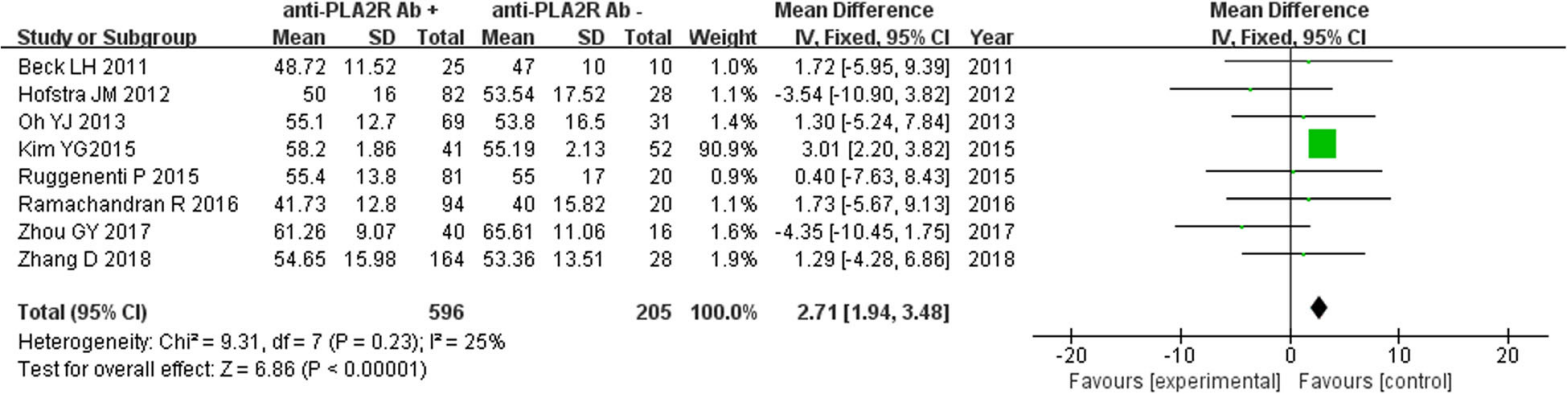
Meta-analysis of age: serum anti-PLA2R antibody-positive group versus -negative group

Eight studies reported the age of IMN patients disaggregated by anti-PLA2R antibody-positive (*n* = 596) and -negative (*n* = 205) groups. Owing to the lack of significant heterogeneity (*P* = 0.23, *I*^*2*^ = 25%), the fixed effect model was used for analysis. The results showed that age of patients in the anti-PLA2R antibody-positive group was significantly higher than that in the anti-PLA2R antibody-negative group [MD = 2.71, 95% CI (1.94, 3.48), *P* < 0.00001] (Fig. 4).

**Fig. 5:**
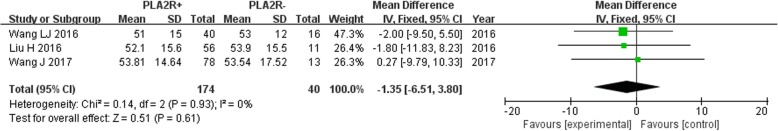
Meta-analysis of age: renal tissue PLA2R-positive group versus -negative group

Three studies reported the age of IMN patients disaggregated by renal tissue PLA2R-positive and -negative groups. Owing to the lack of significant heterogeneity among the studies (*P* = 0.93, *I*^*2*^ = 0%), the fixed effect model was used. The results showed no significant difference between the age of patients in the two groups [MD = -1.35, 95% CI (− 6.51, 3.80), *P* = 0.61] (Fig. 5).

### Comparison of serum creatinine level between anti-PLA2R antibody -positive and -negative groups

**Fig. 6:**
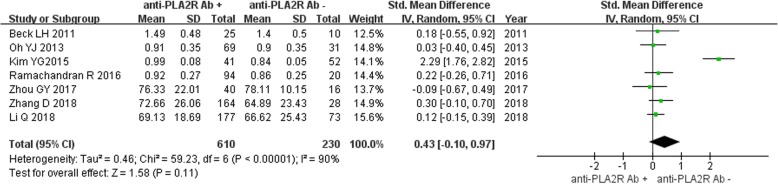
Meta-analysis of serum creatinine: serum anti-PLA2R antibody-positive group versus -negative group

Seven studies reported serum creatinine level in IMN patients disaggregated by serum anti-PLA2R antibody -positive (*n* = 610) and -negative (*n* = 230) groups. Owing to the lack of significant heterogeneity (*P* < 0.0001, *I*^2^ = 90%), the fixed-effect model was used for analysis. There was no significant difference between the two groups [SMD = 0.43, 95%CI (− 0.10, 0.97), *P* = 0.11] (Fig. 6).

### Comparison of estimated glomerular filtration rate between anti-PLA2R antibody -positive and -negative groups

**Fig. 7:**
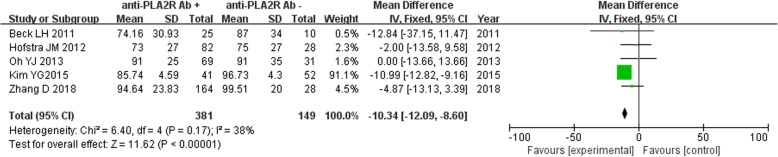
Meta-analysis of eGFR: serum anti-PLA2R antibody-positive group versus -negative group

Five studies reported the estimated glomerular filtration rate (eGFR) of IMN patients disaggregated by serum anti-PLA2R antibody -positive (*n* = 381) and -negative (*n* = 149) groups. Owing to the lack of significant heterogeneity (*P* = 0.17, *I*^2^ = 38%), the fixed effect model was used. eGFR of patients in the serum anti-PLA2R antibody -positive group was significantly lower than that in the serum anti-PLA2R antibody-negative group [MD = -10.34, 95% CI(− 12.09, − 8.60), *P* < 0.00001] (Fig. 7).

### Comparison of 24-h urine protein between serum anti-PLA2R antibody -positive and -negative groups

**Fig. 8:**
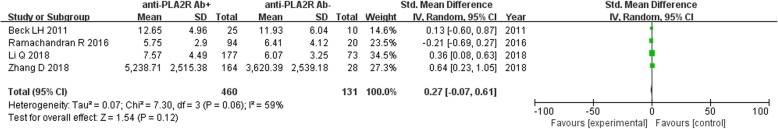
Meta-analysis of 24-h urine protein: serum anti-PLA2R antibody-positive group versus -negative group

Four studies had reported 24-h urinary protein of IMN patients disaggregated by serum anti-PLA2R antibody -positive (*n* = 460) and -negative (*n* = 131) groups. Owing to significant heterogeneity among the studies (*P* = 0.06, *I*^2^ = 59%), the random effect model was used for analysis. The results showed no significant between-group difference in this respect [SMD = 0.27, 95% CI (− 0.07, 0.61), *P* = 0.12] (Fig. 8).

### Comparison of clinical parameters between renal tissue PLA2R -positive and -negative groups

We also performed a meta-analysis of serum creatinine, 24-h urine protein, and eGFR in the renal tissue PLA2R-positive and -negative groups. The 95% confidence interval included 0, and *P* > 0.05. No significant between-group differences were observed in this respect (Table 2).

**Table 2:** Comparison of clinical parameters in renal tissues PLA2R-positive and -negative groups

Parameter	Included studies	Model	PLA2R+(n)	PLA2R-(n)	*P*#	I2	MD/SMD	95%CI	z	P*
eGFR	2	Fixed	96	27	0.45	0%	8.08	-1.53, 17.70	1.65	0.1
Serum creatinine	2	Random	96	27	0.88	0%	-6.33	-16.45, 3.80	1.22	0.22
24-hour urine protein	2	Random	96	27	0.58	0%	0.7	-0.74, 2.13	0.95	0.34

#*P* value for heterogeneity test;*P value for statistical significance.

*MD* mean difference, *SMD* standardized mean difference, *CI* confidence interval, *eGFR* estimated glomerular filtration rate

### Comparison of remission rate between anti-PLA2R antibody, renal tissue PLA2R -positive and -negative groups

**Fig. 9:**
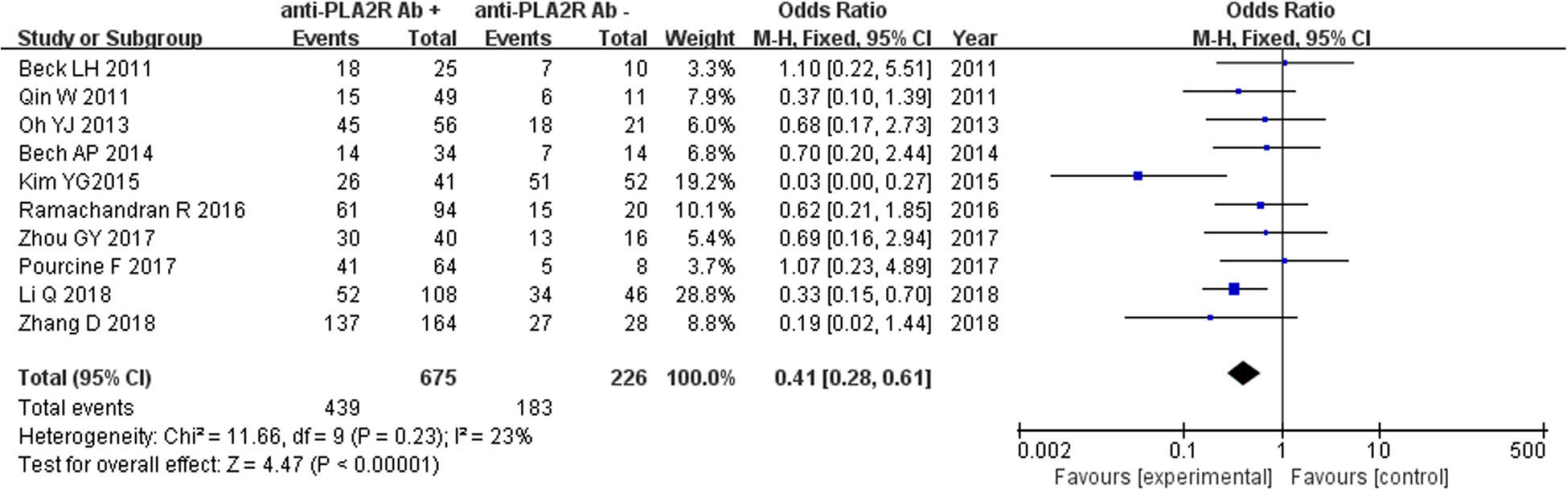
Meta-analysis of remission rate: serum anti-PLA2R antibody-positive group versus -negative group

Ten studies reported the remission rate of IMN patients disaggregated by serum anti-PLA2R antibody -positive (*n* = 675) and -negative (*n* = 226) groups. Owing to the lack of significant heterogeneity among the studies (*P* = 0.23, *I*^2^ = 23%), the fixed effect model was used. The results of the meta-analysis were *z* = 4.47, *P* < 0.00001, and the difference was statistically significant (Fig. 9). The combined effect of OR was 0.41, 95% CI: (0.28, 0.61). When OR = 1.00, it indicated that serum anti-PLA2R antibody -positive status was not related to the rate of remission in IMN patients. When OR > 1.00, it indicated that anti-PLA2R antibody -positive status was related to the remission rate of IMN patients, and the remission rate was high for these patients. When OR < 1.00, it indicated that anti-PLA2R antibody -positive status was related to the remission rate, but had low remission rate. When OR was more distant from 1.00, it indicated greater strength of correlation. If the invalid value (1.00) is within 95% CI, it indicated the lack of statistical significance. In this analysis, the invalid value was not within 95% CI, but was farther away from the original value, which suggested that the expression of serum anti-PLA2R antibody reduced the remission rate of IMN patients with increase in the strength of correlation.

**Fig. 10:**
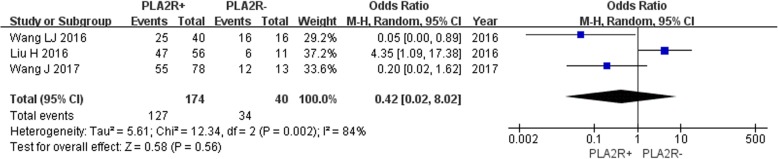
Meta-analysis of the remission rate: renal tissue PLA2R-positive group versus -negative group

Three studies reported the complete remission rate of IMN patients disaggregated by renal tissue PLA2R -positive and -negative groups. Owing to the lack of significant heterogeneity among the studies (*P* = 0.002, *I*^2^ = 84%), the fixed effect model was used. The results showed no significant difference in the remission rate between the two groups [OR = 0.42, 95% CI (0.02, 8.02), *P* = 0.56] (Fig. 10).

### Relationship of remission rate and titre levels of serum anti-PLA2R antibody

**Fig. 11:**
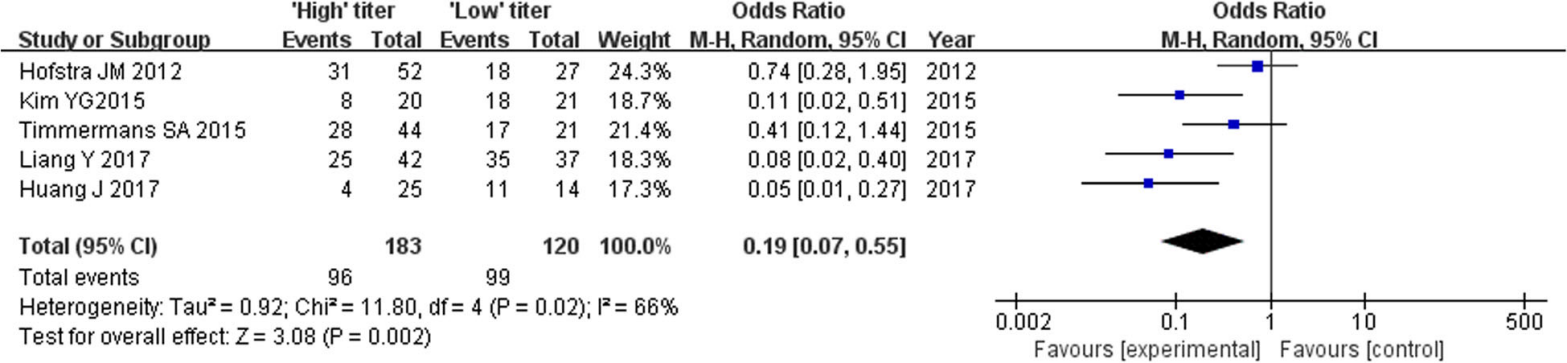
Meta-analysis of remission rate: high-titer versus low-titer subgroups of the serum anti-PLA2R antibody -positive group

Five studies reported remission rates of serum anti-PLA2R antibody-positive IMN patients disaggregated by high-titer (*n* = 183) and low-titer (*n* = 120) groups. Owing to significant heterogeneity among the studies (*P* = 0.02, *I*^2^ = 66%), the random effect model was used. The results of the meta-analysis were *z* = 3.08, *P* = 0.002 < 0.05, and the difference was statistically significant (Fig. 11). The combined effects were OR = 0.19 (95% CI: 0.07, 0.55). The invalid value was not within the 95% CI, and the difference was statistically significant. Away from 1.00, suggesting that the high expression of serum anti-PLA2R antibody reduced the remission rate of IMN patients and the strength of correlation was greater.

### Comparison of adverse prognosis of IMN patients with serum anti-PLA2R antibody positive and negative groups

**Fig. 12:**
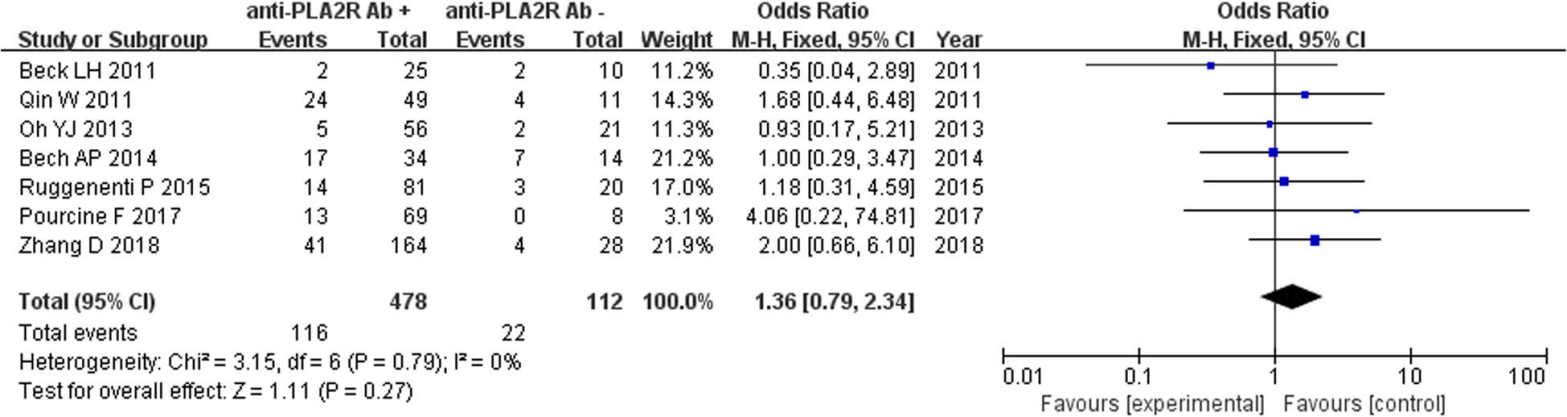
Meta-analysis of adverse prognosis: serum anti-PLA2R antibody-positive group versus -negative group

Seven studies reported the occurrence of adverse prognosis in 590 IMN patients with serum anti-PLA2R antibody positive (*n* = 478) and negative (*n* = 112) groups. Owing to the lack of significant heterogeneity among the studies (*P* = 0.79, *I*^2^ = 0%), the fixed effect model was used. The meta-analysis results showed that *z* = 1.11, *P* = 0.27 > 0.05, and the difference was not statistically significant (Fig. 12). The combined effect was OR = 1.36, 95% CI: 0.79, 2.34, and the invalid value was 1.00 within 95% CI. The study suggested that there was no significant difference in the incidence of poor prognosis between the two groups of IMN patients.

### Comparison of adverse prognosis in the high-titer and low-titer anti-PLA2R antibody -positive subgroups

**Fig. 13:**
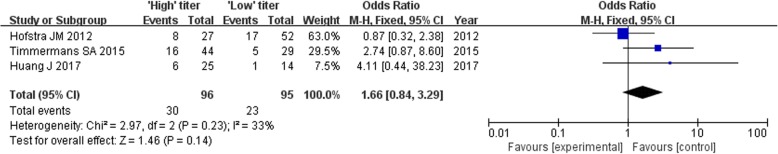
Meta-analysis of adverse prognosis: high-titer versus low-titer subgroup of the serum anti-PLA2R antibody -positive group

Three studies reported the occurrence of adverse prognosis in anti-PLA2R antibody-positive IMN patients disaggregated by high-titer (*n* = 96) and low-titer (*n* = 95) subgroups. Owing to significant heterogeneity among the studies (*P* = 0.23, *I*^2^ = 33%), the random effect was used. The results of meta-analysis were z = 1.46, *P* = 0.14 > 0.05, and the difference was not statistically significant (Fig. 13). The combined effects were OR 1.66, 95% CI: 0.84, 3.29, and the invalid value was within 95% CI. There was no significant difference in the incidence of adverse prognosis between the two sub-groups of anti-PLA2R antibody positive IMN patients.

### Assessment of publication bias

We also assessed publication bias using Egger’s test and Begg’s Test with Stata 13.0 software (Table 3). The results showed that for the comparison of serum anti-PLA2R antibody -positive and -negative groups, except for the age, all other factors were not statistically significant (*P* > 0.05); this suggested the absence of publication bias. For the comparison of serum anti-PLA2R antibody high- and low -titer subgroups, except for the remission rate, all other factors were not statistically significant (*P* > 0.05), which suggested that there was no publication bias. Comparison of serum albumin in serum anti-PLA2R antibody -positive and -negative groups and comparison of the remission rate in serum anti-PLA2R antibody high- and low -titer subgroups were associated with *P* value < 0.05, which was indicative of the presence of publication bias.

**Table 3:** Analysis of publication bias

Factors		Egger's Test			Begg’s Test
		t	*P*	%95CI	*P*r > |z|
Serum albumin	serum	-2.36	0.056	-19.94,0.34	0.035
	renal tissues	0.07	0.958	-67.77, 68.49	1
Age	serum	-2.93	0.026	-1.93,-0.17	1
	renal tissues	-0.25	0.843	-60.93, 58.56	1
Serum creatinine	serum	0.66	0.539	-8.16,13.79	0.386
eGFR	serum	2.26	0.109	-0.50,2.94	0.462
24-hour urine protein	serum	-0.61	0.602	-13.2,9.91	0.734
Remission rate	serum	-0.25	0.809	-3.26,2.63	1
	renal tissues	-4.44	0.141	-24.65, 11.88	0.296
	higher or low titer group	-9.2	0.003	-9.94, -4.83	0.027
Adverse effects rate	serum	-0.43	0.684	-3.29,2.34	0.548
	higher or low titer group	0.86	0.549	-30.83, 35.29	1

*CI* confidence interval, *eGFR* estimated glomerular filtration rate

## Discussion

IMN is the most common pathological type of nephrotic syndrome in the elderly people [28], and it is one of the major underlying causes of ESRD [2]. With the progress in understanding of its pathogenesis, IMN is now recognized as an autoimmune disease [29]. The clinical diagnosis of IMN mainly relies on renal biopsy. The patient’s condition and treatment effect are evaluated mainly by monitoring 24-h urine protein, serum albumin, and serum creatinine [30]. Although renal biopsy is the gold standard for diagnosis of IMN, the procedure is associated with a risk of bleeding and infection. Identification of relatively safe and convenient method for diagnosis and prognostic assessment of patients with IMN will confer a considerable leverage to nephrologists. As early as 2002, Debiec et al. [31] found that neutral endopeptidase is the target antigen of podocytes in membranous nephropathy; however, it is found in only a small proportion of IMN patients. A large number of studies have demonstrated the diagnostic relevance of serum anti-PLA2R antibodies in the context of IMN. In a meta-analysis [32], serum anti-PLA2R antibodies exhibited 68% sensitivity and 97% specificity for the diagnosis of IMN. A large body of evidence supports the relationship between PLA2R and the clinical course and prognosis of IMN. Wang J et al. [11] followed up 91 patients with pathologically confirmed IMN for 15 months. These included 78 serum anti-PLA2R antibody -positive patients and 13 serum anti-PLA2R antibody -negative patients; all patients were administered immunosuppressive therapy [glucocorticoid plus cyclophosphamide (*n* = 45); glucocorticoid plus calcineurin inhibitors (*n* = 46)]. They found that in the third and sixth month of treatment, the remission rate of serum anti-PLA2R antibody -positive patients was lower than that of -negative patients; in addition, serum anti-PLA2R antibody -negative patients showed a faster response to immunosuppressive therapy as compared to serum anti-PLA2R antibody -positive patients. Pang et al [33] found a positive correlation of serum anti-PLA2R antibody levels with serum albumin, serum creatinine, eGFR, and urinary protein. The glomerular PLA2R deposition intensity showed a weak correlation with proteinuria. They suggested that serum anti-PLA2R antibody more closely reflects the disease activity and renal function as compared to PLA2R. In a study by Wei et al. [34], serum anti-PLA2R antibody and renal tissue PLA2R positivity rates in IMN patients were 82.3 and 85.8%, respectively. Serum anti-PLA2R antibody titers were significantly associated with proteinuria in the first 20 months of follow-up. Moreover, changes in serum anti-PLA2R antibody levels preceded the changes in proteinuria. They concluded that serum PLA2R antibody titer, but not renal expression of PLA2R, reflects the prognosis of IMN. Studies [35] have also shown that the effect of immunosuppressive therapy on anti-PLA2R antibody titer in the first 3 months of treatment is a sensitive and specific predictor of therapeutic response at 6th and 9th month of treatment in patients with IMN; further, decrease in antibody at the 6th month was also shown to predict the response at the 12th month. These studies further suggest that serum anti-PLA2R antibodies and PLA2R are closely related to the disease activity and prognosis of IMN.

The present meta-analysis addresses a gap in contemporary literature in that this is the first meta-analysis of the clinical relevance and prognostic value of serum anti-PLA2R antibodies and renal expression of PLA2R in patients with IMN. The results showed that serum albumin and eGFR in serum anti-PLA2R antibody -positive group of IMN patients were lower than those in the serum anti-PLA2R antibody-negative group, while age and serum creatinine levels were higher in the former group. No significant between-group difference was observed with respect to 24-h urine protein. No significant difference was observed between renal tissue PLA2R -positive and -negative groups with respect to age, 24-h urine protein, eGFR, or serum albumin. In summary, serum anti-PLA2R antibody expression may correlate with the severity of IMN. The endpoints were sparsely including the serum albumin levels (Fig. 3), 24-h urine protein (Fig. 3) and the remission rate (Fig. 10) in our study, but they are 3 important index during the treatment of membranous nephropathy, which are used to reflect the severity of membranous nephropathy. Age (Fig. 5) and adverse prognosis (Fig. 13) are 2 established prognostic factors in membranous nephropathy. So, the above indicators are essential in our meta-analysis. Our findings are consistent with those of several previous clinical studies [33, 34], although some findings were different from other studies [33]. These may be attributable to differences with respect to the nature of research, objective, measurement technology used and clinical treatment methods. Due to inconsistent clinical data included in the study, we were unable to compare other factors such as blood lipids and blood pressure. For prognostic assessment of IMN patients, we found that serum anti-PLA2R antibody expression correlated with the remission rate of IMN patients, as the remission rate in the serum anti-PLA2R antibody-positive group was lower than that in the -negative group. Serum anti-PLA2R antibody expression levels were also associated with remission rates The high-titer group had a lower response rate; however, the expression of PLA2R in the renal tissue and the expression level were not significantly associated with remission rate. We did not conduct a meta-analysis about the relationship between the expression of PLA2R in the renal tissue and the poor prognosis of IMN, because only one study was included. However, for the reverse prognosis of IMN patients we did not conduct a meta-analysis because only one study of PLA2R in the renal tissue was included. In addition, the expression of serum anti-PLA2R antibody has no correlation to poor prognosis of IMN. In addition, serum anti-PLA2R antibody expression and the level of expression and IMN patients have no significant statistical significance. Although our meta-analysis results are different from the above results, serum anti-PLA2R antibody expression still has certain guiding significance for monitoring the severity of IMN patients and judging the prognosis. Meta-analysis aims to increase the credibility of conclusions and solve the inconsistency of research results by increasing the sample content. Although some endpoints were sparsely and inconsistently reported by some primary studies, meta-analysis can still increase the credibility of the original literatures and provide more powerful evidence for clinical work. Some limitations of our meta-analysis need to be considered while interpreting our results: (1) only 3 studies had reported data on PLA2R expression in renal tissues, and the sample size of patients was relatively small, which may have affected our results. (2) Lack of relevant data from individual studies may reflect the potential impact of publication bias. (3) The results of individual studies are liable to be influenced by the research subjects, measurement methods, and treatment modalities. Due to a large number of factors, no subgroup analysis was performed. Therefore, the source of heterogeneity among the included studies could not be assessed. (4) Due to the failure to obtain the original data of the included studies, what the specific PLA2R level and serum anti-PLA2R antibody titer was related to adverse prognosis of IMN were not indicated. Due to the failure to obtain the original data of the included studies, the specific PLA2R and serum anti-PLA2R antibody titer values were not indicated when introducing the relationship between PLA2R and its antibodies and IMN conditions. (5) There may be some potential factors in other studies that have not been included, resulting in biased selection in this meta-analysis. (6) We didn’t register our review in PROSPERO.

Future research may be improved in the following respects: (1) Expanding the sample size, designing a multi-center cohort study, and extending the follow-up time as much as possible. (2) For further clarification of the relationship of serum anti-PLA2R antibody and PLA2R titer with clinical course of IMN, it is best to provide a specific titer range.

## Conclusion

As compared to PLAR2, serum anti-PLAR2 antibody is more closely related with iMN disease progression. Compared with negative group, serum anti-PLA2 antibody -positive group had lower serum albumin, higher age, lower eGFR, and lower remission rate. In serum anti-PLA2R antibody -positive group, the high-titer group had lower remission rate. The relationship of PLA2R expression in renal tissue with the clinical manifestations and prognosis of IMN patients cannot be ruled out.

## Data Availability

The datasets used and analysed in the current study are available from the corresponding authors on reasonable request.

## Abbreviations

eGFR: Estimated glomerular filtration rate
ELISA: Enzyme-linked immunosorbent assay
ESRD: End stage renal disease
GBM: Glomerular basement membrane
IFA: Indirect immunofluorescence assay
IMN: Idiopathic membranous nephropathy
MD: Mean difference
MN: Membranous nephropathy
NOS: Newcastle-Ottawa scale
OR: Odds ratio
PLA2R: M-type phospholipase A2 receptors
RCTs: Randomized controlled trials
RR: Relative risk
SMD: Standardized mean difference
WB: Western blotting
